# Profile of chronic kidney disease modifiable risk factors in a rural community of south east Nigeria

**DOI:** 10.1186/s12889-018-5603-6

**Published:** 2018-07-27

**Authors:** Adaobi I. Bisi-Onyemaechi, Henrietta U. Okafor, Maduka D. Ughasoro

**Affiliations:** Department of Paediatrics, College of Medicine, University of Nigeria Ituku-Ozallam, Enugu, Nigeria

**Keywords:** Risk factors, Chronic kidney disease, Rural community, Nigeria

## Abstract

**Background:**

Chronic kidney disease (CKD) is on the increase globally. Prevention of this condition is ideal, however early detection of the disease becomes desirable where the disease process has begun as there are known interventions which can slow the progress to end stage renal disease (ESRD). This study aimed at detecting the profile of some modifiable risk factors for CKD in a cohort of household heads in a rural community with limited resources for managing chronic kidney diseases.

**Methods:**

The study was conducted in a rural community in southeast Nigeria. One hundred and forty five household heads from randomly selected households were interviewed. Their blood pressures were taken and their urine tested. The data was analyzed using SPSS version 21. Simple frequencies and means were calculated.

**Results:**

A total of 145 house hold heads were enrolled. Their mean age was 45.08 (19.65) years. Forty-seven percent had no prior knowledge of their blood pressure and 31.5% were found to be hypertensive. Only one study participant (1%) had ever had a urinalysis test and proteinuria and glycosuria were found in 50.4 and 27.9% respectively. Most (75%) patronized patent medicine vendors for their primary healthcare while 31.8% had taken herbal mixtures in the past.

**Conclusion:**

There are presently many modifiable risk factors for the development of chronic kidney disease in rural communities in south-east Nigeria. Urgent targeted intervention is required to forestall an epidemic of CKD in the near future.

## Background

Globally, chronic kidney disease (CKD) prevalence has been noted to be on the increase both in children and adults [1]. Presently it affects 10% of the worldwide population with millions dying due to lack of access to treatment [2]. Worldwide over 2 million people receive treatment in form of dialysis or kidney transplant but this represents only 10% of the people who are dependent on it [3]. In England, according to a recent report published by NHS Kidney Care, chronic kidney disease costs more than breast, lung, colon and skin cancer combined [4]. This will have huge double burden of health and financial implications in places like sub Saharan Africa. There is a further variation in the prevalence of CKD between rural and urban communities [1].The high mortality primarily associated with CKD is further compounded with lack of facilities and widely applied health insurance in resource poor countries.

Presently, there is a strong advocacy towards prevention of CKD achievable through some simple and easily available tools and strategies like routine monitoring of blood pressure, blood sugar and urine for infection. Unfortunately, most people with these CKD predisposing illnesses are not aware that they have these illnesses. Even people who have urinary tract infections (UTI) often take it to be malaria and treated as such [5, 6]. Nevertheless, of all these predisposing illnesses one common finding is detection of protein in urine by simple urinalysis using test strip and this has been identified as one of the most reliable predictor of CKD in its early stage [7, 8].

In view of the huge challenges of management of CKD and potential benefits of surveillance on the early symptoms and signs of the risk factors at the community level, it will be expedient to evaluate the extent to which communities watch out for these indices. In most African communities, the extent of awareness on health as well as their health-seeking behavior of the entire household is determine by the household-head’s level of knowledge and awareness on health related issues [9]. It is usually one of the responsibilities of the house hold head to take decision on health related matters on behalf of other members of the household [10]. There are multitude of challenges that often confront decision making when a member of the household is ill [11, 12]. An evaluation of preventive healthcare being practiced by household heads with regards to rising prevalence of CKD would helpful in the design of preventive strategies and interventions for CKD. Thus in this study the researchers set out to ascertain the depth of information on risk factors for CKD and the prevalence of urinary abnormalities and other known risk factors of CKD amongst household heads in a rural community in Enugu.

## Methods

### Study site

The study was conducted in Mbogodo, a rural community in Nkanu West Local Government Area (LGA) in Enugu South-East Nigeria.

### Study design

This was a cross-sectional descriptive study. A multistage sampling technique was used to select a community in Nkanu-West of Enugu state Nigeria. Study location was Mgbogodo, a rural community with a population of about 30, 000 for both adults and children, with estimated 590 households. There are average annual rainfall of 30 cm and maximum temperature of 35 °C. It has a tropical rain forest vegetation and malaria is holo-endemic in this region. The community is agrarian in nature with few female petty traders. There is a General hospital and a health center, both about 3 km away from the community.

### Sample size

Three hundred and sixty four households were enumerated out of which 305 households were selected randomly to participate in the study. The decision to select 305 households was based on absolute response rate of 87 and 49.3% for the first response and second response reported by Fekete *et al* [13] and, the targeted minimum sample size of 130 household heads. Since the study involved two stages: first, household selection and obtaining consent from the household heads to come to the health facility for the study and, the second step, which involved interview and investigation of the household heads that later presented to the health facility. Thus, 87% of 305 gave 265 households, and 49.3% of 265 produced 130 participants.

### Data collection

The study involved two phases. The first phase was enumeration of the households in the community. The households that participated in the second phase were selected randomly from the list of the enumerated households. This was to ensure even selection and to prevent skewed of households that would participate in the study and give a better representation of the community. The selected households were revisited and researchers then created rapport with heads of the selected households explaining the purpose of the study as well as obtained consent to participate. They were then asked to visit the community health center for the second part of the survey- health facility-based clinical assessment and investigation. Since the survey was a component of a bigger study which involved laboratory investigations and anthropometric measurements, the selected households were requested to visit the health center for other aspects of the study. While in the health center, interviewer- administered semi-structured questionnaire which has both closed and open ended questions was used to obtain information on their age, educational status, knowledge of the symptoms of urinary tract infections, potential causes of kidney injury, prior knowledge of their blood pressure, previous history of having done a urine test, symptoms experienced in their previous illnesses and their health seeking behavior. The respondents were asked to select one major symptoms of UTI from a list of symptoms and the selected option was checked on the questionnaire, while they were asked to list conditions that can cause kidney injury and their responses written down on the questionnaire. They were allowed to mention as much as they could but only correct options were documented. For the urinalysis, The respondents were previously advised to take adequate water just before coming to the health centre. At the health centre, urine was collected from each of them in a universal container. Urinalysis was done immediately with Combi-9 rapid test kit and results were documented in case record forms. Standard practices were observed during blood pressure measurement. The measurement was taken 30 min later to allow adequate time for rest. Two different measurements of the blood pressures of the study participants were taken on the right arm in sitting position using Henson^R^ mercury sphygmomanometer and the average documented subsequently.

### Data management

Data management was done with SPSS software version 21 Chicago Illinois. Frequencies and means of continuous variables were found. Results of urinalysis done with the Combi 9 dipstick and the presence of protein was described in levels; negative, trace, 1+, 2+, and 3+. Proteinuria was taken as “1+ or more” reading on the combi urine test kit. Blood pressure was classified as normal: systolic blood pressure (SBP) 90–119 mmHg or diastolic blood pressure (DBP) 60–79 mmHg, Prehypertension SBP 120-139 mmHg or DBP 80 -89 mmHg and Hypertension (HTN) was defined as SBP greater than 140 mmHg and DBP greater than 90 mmHg according to American Heart Association. Hypertension is further classified into Stage 1 HTN: SBP 140–159 or DBP 90-99 mmHg, Stage 2 HTN: SBP ≥ 160 or DBP ≥ 100 mmHg.

## Results

**Fig. 1:**
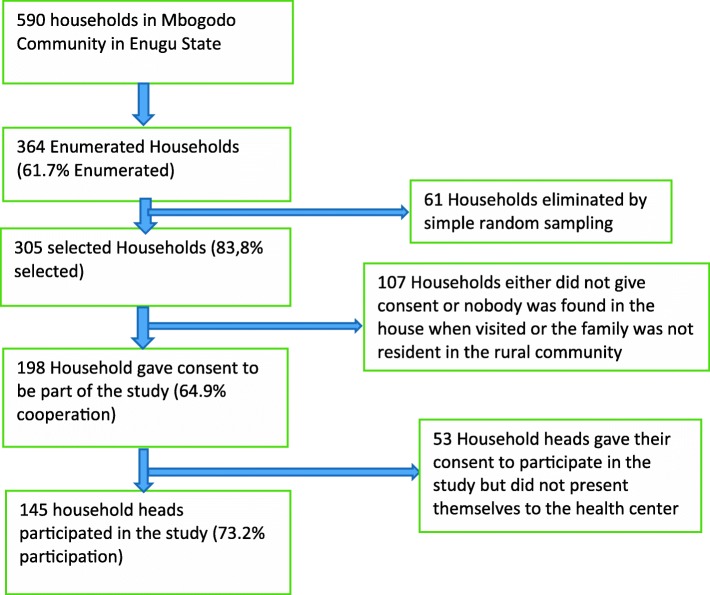
Flow chart representing the selection of the household heads that participated in the study

One hundred and ninety eight household heads from the 305 selected presented to the health center, giving cooperation rate of 64.9% (198/305). A total of 145 household heads responded to the study, giving a consent rate of 73.2% (145/198) (Fig. 1). The mean age of the respondents was 45.08 years. Majority (68.3%) were female. Those with secondary school education and above were 47.6%, (Table 1).

**Table 1:** Socio-demographic Characteristics of the Respondents

Variables	*n* = 145	%
Age in Years		
Mean Age (SD)	45.08 (19.65)	
Gender		
Female	99	68.3
Male	46	31.7
Education		
No formal	38	28.2
Primary	38	26.2
Secondary	50	34.5
Tertiary;(University/College)	19	13.1

Majority (58.6%) of the respondents identified change in urine stream as a symptom of urinary tract infection, all other symptoms collected were only recognized by a small percentage (41.4%) of the respondents. (Table 2) Most mentioned hepatitis (62.1) as a cause of kidney disease while none identified hypertension as a cause of kidney injury.

**Table 2:** The Respondents’ Knowledge of Urinary Tract Infection

Variables	*n*	%
Knowledge of Symptoms of Urinary Tract Infection (*n = 143*)		
Change in Urine Stream	85	59.4
Urethral Discharge	21	14.7
Lower Abdominal Pain	19	13.3
Fever	10	7.0
Change in Urine colour	5	3.5
Dysuria	2	1.4
Back Pain	1	0.7
Vomiting	0	0.0
Knowledge of Potential Causes of Kidney Injury^a^		
Hepatitis	90	62.1
Kidney Injury	31	21.4
Diabetes	26	17.9
Malaria	18	12.4
Urinary Infection	7	4.8
Others	12	8.3

^a^Some respondents gave more than one response

The number of the respondents that had prior knowledge of their blood pressure was 61 (47%.) The prevalence of hypertension among the respondents was 31.5% while 52.8% were pre-hypertensive. (Table 3).

**Table 3:** The Blood Pressure Status of the Respondents

Variables	*n* = 130	%
Do you Know your Blood Pressure (*n* = 127)		
Yes	61	47
No	69	53
Hypertensive based on Sphygmomanometer Measurement (*n* = 130)		
Yes	41	31.5
No	89	68.5
Pre-Hypertensive (*n* = 89)	47	52.8
Hypertension stage I	18	43.9
Hypertension stage 2	18	43.9
Hypertension stage 3	6	12.2

Normal blood pressure: systolic blood pressure (SBP) 90–119 mmHg or diastolic blood pressure (DBP) 60–79 mmHg, Prehypertension: SBP 120-139 mmHg or DBP 80-89 mmHg and Hypertension (HTN): SBP ≥ 140 mmHg and DBP ≥ 90 mmHg. Stage 1 HTN: SBP 140–159 or DBP 90-99 mmHg, Stage 2 HTN: SBP ≥ 160 or DBP ≥ 100 mmHg

Headache was the commonest symptom experienced during the last illness. (Table 4) Majority patronized patent medicine vendors during this illness while one person took herbal concoction. Only one respondent had ever done a urine test previously.

**Table 4:** Health Seeking Behavior of the Respondents in their Previous Illnesses

Variable	*N*	%
Action Taken During Last Illness (*n* = 125)		
Visited Patent Medicine Vendor	94	75.2
Visited Hospital/Doctor	17	13.6
No Treatment Received	10	8.0
Prayed	2	1.6
Laboratory Test	1	0.8
Herbal Therapy	1	0.8
Previous Urine Test (*n* = 98)		
Yes	1	1.0
No	97	99.0
Previous Ingestion of Herbal Concoction (*n* = 129)		
Yes	41	31.8
No	88	68.2

Protein was the most frequent (50.4%) abnormality found in their urine, followed by glucose (27.9%). (Table 5).

**Table 5:** Urinalysis results of the Respondents

Variables	*n*	%
Hematuria (*n = 120*)		
Yes	7	5.8
No	113	94.2
Glucose (*n = 122*)		
Yes	34	27.9
No	88	72.1
Proteinuria (*n = 123*)		
Yes	62	50.4
No	61	49.6
Leukocyte esterase (*n = 100*)		
Yes	13	13.0
No	87	87.0
Nitrite (*n = 121*)		
Yes	5	4.1
No	116	95.9

## Discussion

There was an overall poor knowledge of symptoms attributable to kidney diseases among the respondents. This is similar to what has been reported in previous studies [14, 15]. This finding is not peculiar with kidney diseases; there is an existing dearth of health knowledge even with other illnesses among the rural poor in Nigeria and most developing nations. This can be attributed to low literacy rates and subsequent poor utilization of formal health care services as was observed in this study and previous ones [16–18]. The poor utilization of formal health service in this community could be due to distance of the health facilities to the community whose main mode of transportation is Also, poor flow of health information between health care providers and their patients/clients during clinic visits contributes to low awareness of health issues even among the few privileged to access formal health care. This situation can be improved if there is a functional health system that aids quick and convenient presentation to the health facilities and health care providers inculcating the art of effective health communication and information sharing with their clients.

Among the risk factors evaluated, most of the respondents had no prior knowledge of their blood pressure levels. Similar findings had been reported [19–21], and this is not unexpected in regions where access to formal health care for even overt health problems is low [22, 23]. Antenatal care [24] and immunization services [25] are the only preventive services not underutilized in this environment. Blood pressure measurement is routine for almost every health facility visit, therefore any visit to any tier of health service provider would likely improve this situation. The long term cost of this ignorance would be much considering the relative high prevalence of hypertension documented in this study. Similarly most have never had a urinalysis in the past in the face of high prevalence of asymptomatic proteinuria and glycosuria. This has also been documented in other studies.[26–28] Proteinuria and hypertension are known risk factors for chronic kidney disease. It has been widely shown that proteinuria may accelerate kidney disease progression to end-stage renal failure. Furthermore, glycosuria was detected in an appreciable number of respondents who were not aware that they were more likely to be diabetic. Since glucose appears in urine after a certain high threshold level of blood glucose is reached and well within diabetic range. Underlying the above situations are poverty and ignorance which has perpetuated some kidney risky practices such as use of herbal concoction for the treatment of illnesses as found in this study. The damaging effect of herbal concoction has been widely reported [29–32]. It is urgent to improve knowledge and practices considering the negative impact of the combination of poor knowledge of health status, use of herbal concoction and the high patronage of PMVs. These will eventually lead to late detection of any existing illness.

Patronage of patent medicine vendors for treatment of their illnesses is a common practice in Nigeria and other sub-Saharan African countries [33–35]. Onyeneho et al. [35] noted that 70% of their study participants patronize patent medicine vendors similar to the finding in this study. This practice may result from the belief that hospitals are reserved for “serious illnesses” as reported by some respondents. It may also explain the low prevalence of urinalysis among the study participants as the medicine vendors may lack the capacity to think beyond malaria when presented with certain symptoms. Hospital services include physical examination and laboratory investigations which help detect asymptomatic illnesses therefore affording time for early intervention and better outcomes.

A limitation in this study was the inability to examine the urine and check the blood pressure of other household members especially children. This information would have given insight into the prevalence of deranged urinary indices and hypertension among the paediatric population. Another limitation was, restricting respondents to one major symptom of UTI. They would have been allowed more options or the question left open ended. Expanding the options would have yielded more information on their knowledge on UTI and other symptoms associated with UTI. Furthermore, the lack of details (reasons, source, components, dosing and cost) of the ingestion of herbal concoction is a limitation on this study. With emerging evidence on the contribution of herbal mixtures to incidence of kidney injury, these would have been necessary.

## Conclusion

Knowledge of kidney related symptoms was poor among the residents of Mgbogodo Southeast Nigeria. Majority had never done a urine test in the past. There was a high prevalence of high blood pressure in the population of which most were ignorant of. The services of patent medicine vendors were highly solicited in times of ill health. These are high risk factors for development of chronic kidney disease however they are all modifiable. Community health awareness and education programs may help in improving the health seeking behavior of this rural community to reduce or totally prevent the a rising prevalence of a possible CKD in future.

## Abbreviations

CKD: Chronic kidney disease
ESRD: End stage renal disease
LGA: Local government area
NHS: National health service
SD: Standard deviation
SPSS: Statistical package for social sciences
UTI: Urinary tract infection
